# COPD profiles and treatable traits using minimal resources: identification, decision tree and stability over time

**DOI:** 10.1186/s12931-022-01954-6

**Published:** 2022-02-14

**Authors:** Alda Marques, Sara Souto-Miranda, Ana Machado, Ana Oliveira, Cristina Jácome, Joana Cruz, Vera Enes, Vera Afreixo, Vitória Martins, Lília Andrade, Carla Valente, Diva Ferreira, Paula Simão, Dina Brooks, Ana Helena Tavares

**Affiliations:** 1 Lab3R – Respiratory Research and Rehabilitation Laboratory, School of Health Sciences, University of Aveiro (ESSUA), Agras do Crasto – Campus Universitário de Santiago, Edifício 30, 3810-193 Aveiro, Portugal; 2 iBiMED – Institute of Biomedicine, Department of Medical Sciences, University of Aveiro, Agras do Crasto – Campus Universitário de Santiago, Edifício 30, 3810-193 Aveiro, Portugal; 3 School of Rehabilitation Science, McMaster University, Hamilton, Canada; 4 West Park Healthcare Centre, Toronto, Canada; 5 Center for Health Technology and Services Research (CINTESIS) and Department of Community Medicine, Information and Health Decision Sciences (MEDCIDS), Faculty of Medicine, University of Porto, Porto, Portugal; 6 Centre for Innovative Care and Health Technology (ciTechCare), Polytechnic of Leiria, Leiria, Portugal; 7 Center for Research & Development in Mathematics and Applications (CIDMA), University of Aveiro, Aveiro, Portugal; 8 Pulmonology Department, Hospital Distrital Figueira Foz, Figueira da Foz, Portugal; 9 Pulmonology Department, Centro Hospitalar do Baixo Vouga, Aveiro, Portugal; 10 Pulmonology Department, Centro Hospitalar do Médio Ave, Famalicão, Portugal; 11 Pulmonology Department, Unidade Local de Saúde de Matosinhos, Matosinhos, Portugal

**Keywords:** Phenotype, Cluster analysis, Treatable traits, Decision trees, COPD

## Abstract

**Background and objective:**

Profiles of people with chronic obstructive pulmonary disease (COPD) often do not describe treatable traits, lack validation and/or their stability over time is unknown. We aimed to identify COPD profiles and their treatable traits based on simple and meaningful measures; to develop and validate a decision tree and to explore profile stability over time.

**Methods:**

An observational, prospective study was conducted. Clinical characteristics, lung function, symptoms, impact of the disease (COPD Assessment Test—CAT), health-related quality of life, physical activity, lower-limb muscle strength and functional status were collected cross-sectionally and a subsample was followed-up monthly over six months. A principal component analysis and a clustering procedure with k-medoids were applied to identify profiles. A decision tree was developed and validated cross-sectionally. Stability was explored over time with the ratio between the number of timepoints that a participant was classified in the same profile and the total number of timepoints (i.e., 6).

**Results:**

352 people with COPD (67.4 ± 9.9 years; 78.1% male; FEV_1_ = 56.2 ± 20.6% predicted) participated and 90 (67.6 ± 8.9 years; 85.6% male; FEV_1_ = 52.1 ± 19.9% predicted) were followed-up. Four profiles were identified with distinct treatable traits. The decision tree included CAT (< 18 or ≥ 18 points); age (< 65 or ≥ 65 years) and FEV_1_ (< 48 or ≥ 48% predicted) and had an agreement of 71.7% (Cohen’s Kappa = 0.62, p < 0.001) with the actual profiles. 48.9% of participants remained in the same profile whilst 51.1% moved between two (47.8%) or three (3.3%) profiles over time. Overall stability was 86.8 ± 15%.

**Conclusion:**

Four profiles and treatable traits were identified with simple and meaningful measures possibly available in low-resource settings. A decision tree with three commonly used variables in the routine assessment of people with COPD is now available for quick allocation to the identified profiles in clinical practice. Profiles and treatable traits may change over time in people with COPD hence, regular assessments to deliver goal-targeted personalised treatments are needed.

**Supplementary Information:**

The online version contains supplementary material available at 10.1186/s12931-022-01954-6.

## Background

Chronic obstructive pulmonary disease (COPD) is highly heterogeneous and complex hence, personalising assessments and treatments to this population across different settings and available resources imposes challenges and debate [1, 2].

Research efforts have been made to advance knowledge in this field, namely through the identification of homogeneous subgroups of patients with COPD [3, 4], the so-called clinical phenotypes or profiles, grouped by different type of personal characteristics (e.g., genetic, clinical, biochemical, radiological) for prognostic and therapeutic purposes [3–12]. More recently, a new approach, “treatable traits”, i.e., pulmonary, extra-pulmonary and behaviour/lifestyle characteristics of each person that are clinically relevant, identifiable and treatable, emerged [13–15]. Although studies on clinical profiles and treatable traits have been conducted, their cross-sectional nature, narrow eligibility criteria, main focus on physiological/pulmonary measures often not available across settings [5, 6], absence of decision trees and lack of validation with independent samples [3], limits our understanding of the heterogeneous manifestations of COPD and hinders their applicability in daily clinical practice.

Few studies have tried to identify profiles and their treatable traits using clinical and patient-reported outcomes beyond pulmonary measures and understand their behaviour over time [9, 14, 15]. Moreover, to be easily applied and useful in clinical practice, these profiles need predicting tools, such as decision trees [5]. Simple and validated decision trees are scarce but important as a first line approach, as they quickly enable the allocation of each individual into a profile [5, 10]. Such allocation can then aid the decision-making of the most suitable comprehensive assessments to identify each person’s treatable traits and guide tailored and multicomponent interventions that can alter the course/impact of the disease and its daily management [10, 13, 14].

Thus, we aimed to (i) identify and describe profiles and respective treatable traits in people with COPD based on simple and meaningful clinical measures possible to be collected with minimal resources; (ii) develop and validate a decision tree to quickly identify the profile of each person and (iii) assess the stability of the profiles during a six-months period.

## Methods

### Study design

An observational, prospective cohort study was conducted with data collected between 2017 and 2020 in GENIAL (PTDC/DTP-PIC/2284/2014) and PRIME (PTDC/SAU-SER/28806/2017) projects. PRIME builds on GENIAL project, i.e., settings, recruitment strategies and data collection protocols were similar. Data were first gathered from both projects and then analysed. The study was approved by five Ethics Committees (CHMA 09/2016-10/2018; ULS Matosinhos 10/CES/JAS 17/02/2017-73/CE/JAS 12/10/2018; CHBV 777,638–086,892; HDFF 1807/2017-27/05/2019; ARSCentro 64/2016-85/2018). This work is described according to the Strengthening the Reporting of Observational Studies in Epidemiology (STROBE) statement [16].

### Participants

People with COPD diagnosed according to the GOLD criteria [17], clinically stable in previous month (i.e., no hospital admissions, acute exacerbations or changes in medication) were included. Exclusion criteria comprised the presence of other respiratory diseases or any clinical condition that precluded participation in the assessment (i.e., signs of cognitive impairment or presence of a significant cardiovascular, neurological, musculoskeletal, immunological or infectious disease). Eligible participants were identified in hospitals and primary healthcare centres routine appointments by their clinicians, who explained the study. Interested participants were contacted by the researchers who obtained written informed consents and performed data collection.

### Data collection

Sociodemographic (age, sex), anthropometric (height and weight to compute body mass index [BMI]) and general clinical (smoking habits, self-reported medication for COPD, use of long-term oxygen therapy and non-invasive ventilation, number of acute exacerbations and hospitalisations in the past year) data were collected. Severity of comorbid diseases was scored with the Charlson Comorbidity Index (CCI) and interpreted as: (i) mild, 1–2; (ii) moderate, 3–4; and (iii) severe, ≥ 5 [18]. Self-reported physical activity was assessed with the brief physical activity assessment tool (BPAAT) [19]. Scores range from 0 to 8 being interpreted as 0–3 ‘insufficiently active’ or ≥ 4 ‘sufficiently active’ [19].

Lung function was assessed with a spirometer (MicroLab 3535, CareFusion, Kent, UK) and values from the forced expiratory volume in one second (FEV_1_) percentage predicted were used to classify participants’ according to the GOLD grades (1, 2, 3 and 4) [17]. GOLD groups (A, B, C and D) were determined using the number of acute exacerbations and hospitalisations in the previous year and the COPD assessment Test (CAT) [17].

Impact of the disease was assessed with CAT [20]. Scores range from 0 to 40 and are interpreted as ≤ 10 low, 11–20 medium, 21–30 high and 31–40 very high impact [20]. Activity-related dyspnoea was assessed with the modified British Medical Research Council dyspnoea questionnaire (mMRC) [21]. Scores range from 0 (no trouble with breathlessness) to 4 (too breathless to leave the house) [21].

Symptoms of anxiety and depression were measured with the Hospital Anxiety and Depression Scale (HADS) [22]. Scores range from 0 (minimum symptom load) to 21 (maximum symptom load) [22].

The Saint George’s Respiratory Questionnaire (SGRQ) was used to measure health-related quality of life [23]. Scores range from 0 (no impairment) to 100 (worst possible health-related quality of life) [23].

Quadriceps muscle strength (QMS) was measured through a maximum voluntary isometric contraction with a handheld dynamometer [24] (microFET2, Hoggan Health, The best Salt Lake City, Utah). Measurements were taken at the dominant side [24], and the best of three measurements (less than 10% of variation) was used for analysis. Functional status was measured with the 1-min sit-to-stand test (1minSTS) twice and the best performance was used for analysis [25]. Percentage of predicted for QMS and 1minSTS values was calculated based on reference values [24, 26].

These measures were chosen for their simplicity, wide availability, quick application in space constrained settings and easy identification of evidence-based treatable traits possible to tackle with non-pharmacological interventions, since our population was already receiving standard pharmacological treatment.

Data were collected during an initial visit by trained physiotherapists with more than three years of experience in similar data collection protocols. A subsample of participants was followed-up monthly over six months. Participants of different GOLD groups and grades, living within 100 km of our research facilities and willing to receive a physiotherapist for a short-monthly assessment at their homes were invited to participate. Data collected over the follow-up period consisted of number of acute exacerbations, hospitalisations and changes in medication in the previous month, CAT, mMRC, QMS and 1minSTS.

### Statistical analysis

All analyses were performed in R (version 3.6.1) with a level of significance set at p < 0.05. Normality of data distribution was tested with the Shapiro–Wilk test. Comparison between participants’ characteristics and between the total and follow-up sample were analysed using t-test or Mann Whitney U-test, for continuous variables; and chi-squared or Fisher’s test, for categorical variables. Differences among profile characteristics were analysed using univariate ANOVA followed by Tukey’s multiple comparison test or Kruskal–Wallis test followed by Dunn’s multiple comparison test, for continuous variables; and chi-squared or Fisher’s test, for categorical variables, according to their assumptions.

### Profiles and treatable traits

A principal component analysis was conducted to avoid strongly correlated variables dominating the cluster assignment [27]. Scores associated to the principal components yield a data matrix, on which the clustering algorithm, k-medoids, was applied [28]. The optimal number of clusters, k, was identified using the gap statistic [29] (Additional file). Age, BMI, pack years, CCI, FEV_1_, forced vital capacity (FVC) percentage predicted, CAT, mMRC, HADS-A, HADS-D, SGRQ, QMS, and 1minSTS were explored.

The presence/absence of treatable traits was considered as described in Table 1.

**Table 1:** Treatable traits, measurement instrument and cut-off values used for interpretation in people with chronic obstructive pulmonary disease

Treatable traits	Measurement instrument	Cut-off used for interpretation
Pulmonary traits		
Mild airflow obstruction [17]	FEV_1_%pred [17]	⩾80%pred [17]
Moderate airflow obstruction [17]	FEV_1_%pred [17]	50⩽FEV_1_ < 80%pred [17]
Severe airflow obstruction [17]	FEV_1_%pred [17]	30% ⩽FEV_1_ < 50%pred [17]
Very severe airflow obstruction [17]	FEV_1_%pred [17]	< 30%pred [17]
Frequent exacerbations	Clinical history in the previous year [17]	≥ 2 exacerbations or ≥ 1 hospitalisation in the previous year [17]
Extra-pulmonary traits—physical		
Poor nutritional status	BMI [54]	BMI < 21 or BMI > 30 kg/m^2^ [54]
Lower-limb muscle dysfunction	QMS measured with the HHD [24]	< 70% of percentage predicted [24, 55]
Low functional status	1minSTS [25]	< 70% of percentage predicted [25, 55]
Extrapulmonary traits—symptoms and health status		
Activity-related dyspnoea	mMRC [21]	≥ 2 points [2, 10, 14, 17]
Impact of the disease	CAT [20]	≥ 10 points [10, 14, 17] ≥ 18 points [2]
Symptoms of anxiety	HADS [22]	≥ 8 points in HADS-A [22]
Symptoms of depression	HADS [22]	≥ 8 points in HADS-D [22]
Impact on health-related quality of life	SGRQ [23]	≥ 25 points [17] ≥ 46 points [2]
Extrapulmonary traits—behavioural/life-style risk factors		
Current smoking	Clinical history [17]	Positive at the moment of assessment
Physical inactivity	Brief physical activity assessment tool (BPAAT)	≤ 3 points [19]

*BMI* body mass index, *BPAAT* Brief physical activity assessment tool, *CAT* COPD Assessment Test, *FEV*_*1*_*%pred* FEV_1_, forced expiratory volume in 1 s percentage predicted, *HADS* The Hospital Anxiety and Depression Scale HADS-A anxiety subscale and HADS-D depression subscale, *HHD* HandHeld Dynamometry, *mMRC* Modified British Medical Research Council questionnaire, *QMS* Quadriceps muscle strength, *SGRQ* Saint George’s Respiratory Questionnaire, *1minSTS* 1-min Sit-to-Stand Test. We used two cut-offs for CAT (≥ 10 points and ≥ 18 points) and SGRQ (≥ 25 points and ≥ 46 points): the ones currently recommended^17^ and the ones found to better discriminate burden of symptoms in COPD^2^.

### Decision tree

Random Forest was used to rank the importance of the above-mentioned 13 variables used in the clustering procedure (Additional file). The top variables were selected to construct the decision tree.

The decision tree was developed and validated with two independent data sets randomly selected from the total sample; 70% was used for generation and 30% for validation, i.e., to determine its prediction ability in identifying the correct profile [30]. The accuracy, defined as the proportion of correct classifications, was used to quantify the predictive ability of the decision tree. The decision tree that maximised accuracy was chosen.

Agreement between the profile predicted by the decision tree and the profile defined by the clustering procedure was determined using Cohen’s Kappa and interpreted as: poor (k < 0), slight (0.00 ≤ k ≤ 0.20), fair (0.21 ≤ k ≤ 0.40), moderate (0.41 ≤ k ≤ 0.60), substantial (0.61 ≤ k ≤ 0.80) or almost perfect (k > 0.80) [31].

### Stability of the profiles over time

The decision tree was applied in all timepoints for each participant. Stability of the profiles was assessed with a stability score defined as the ratio between the number of timepoints that a participant was classified in the same profile (most frequent profile for that participant) and the total number of timepoints (i.e., 6). Possible scores ranged from 1/3 (maximum instability—migrating across the 4 profiles) to 1 (complete stability—there was no change in the profile allocation over time).

## Results

**Fig. 1:**
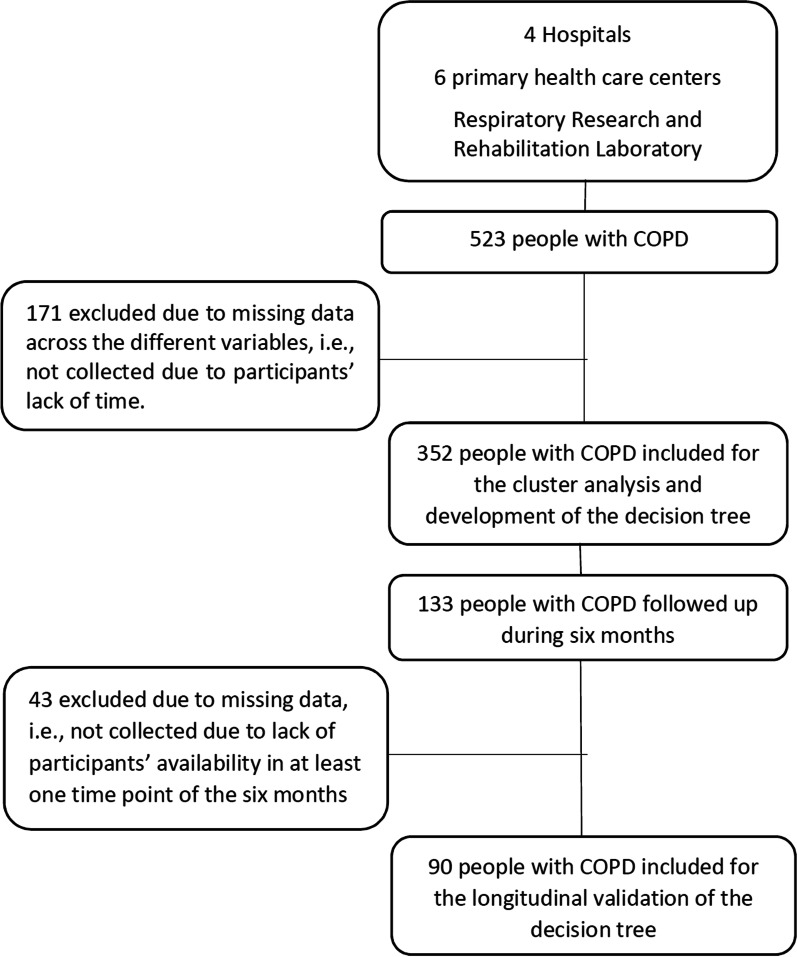
Flow chart of participants’ recruitment for establishing the profiles and developing and validating the decision tree (n = 352) as well as for studying the stability of the proposed profiles (n = 90) in people with chronic obstructive pulmonary disease (COPD)

A total of 523 people with COPD were recruited however, 171 had incomplete data due to participants’ lack of time. Therefore, for establishing the profiles and developing the decision tree, data from 352 people with COPD (67.4 ± 9.9 years; 275[78.1%] male; FEV_1_ = 56.2 ± 20.6% predicted), were used. From these, 133 people were followed monthly for six months. In 43 participants, data were missing in at least one timepoint and therefore, they were excluded from the follow-up analysis. Hence, stability of the profiles was performed with data from 90 people with COPD (67.6 ± 8.9 years old; 77 [85.6%] male; FEV_1_ = 52.1 ± 19.9% predicted). Figure 1 shows the flow of participants in the study.

**Fig. 2:**
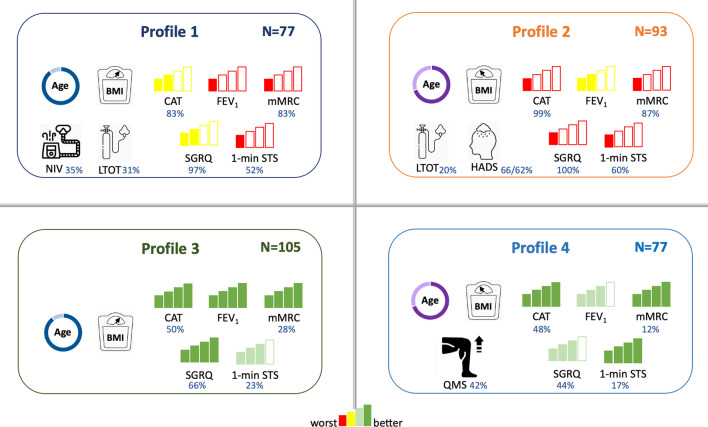
Graphical representation of the four profiles of people with COPD (n = 352) identified. A colour grade system of red (worst), yellow and green (better) was used to facilitate the clinical interpretation of each variable across profiles. Percentage of people on non-invasive ventilation (NIV), long-term oxygen therapy (LTOT), with anxiety/depression symptoms or with impairment are indicated in blue. *BMI* body mass index, *CAT* COPD Assessment Test, *FEV*_*1*_ forced expiratory volume in 1 s, *HADS-A and HADS-D* The Hospital Anxiety and Depression Anxiety and Depression Subscales, *LTOT* Long-Term Oxygen Therapy, *mMRC* Modified British Medical Research Council questionnaire, *NIV* Non-Invasive Ventilation, *QMS* Quadriceps muscle strength, *SGRQ* Saint George’s Respiratory Questionnaire, *1minSTS* 1-min Sit-to-Stand Test

Four profiles were identified (Fig. 2 and Additional file 1: Figure S1). Table 2 presents baseline characteristics and treatable traits for the total sample, per profile and for the longitudinal subsample. Each profile incorporated people with COPD from all GOLD grades and groups (Additional file 1: Figure S2). Profiles 1 and 3 were composed of older, mostly overweight/obese and comorbid people. Profiles 2 and 4 included younger and underweight/normal weight people.

**Table 2:** Characteristics of people with chronic obstructive pulmonary disease (COPD), for the total sample (n = 352), for each of the four profiles identified and for the longitudinal subsample (n = 90)

	Total sample (n = 352)	Profile 1 (n = 77)	Profile 2 (n = 93)	Profile 3 (n = 105)	Profile 4 (n = 77)	p-value (comparisons across profiles)	Longitudinal subsample (n = 90)	p-value (longitudinal vs. total sample)
Age, years	68 (61–74)	71 (67–78)^b,d^	66 (58–71)^a,c,d^	72 (69–78)^b,d^	59 (54–64)^a,b,c^	< 0.001	68 (62–72)	0.945
Sex, n (%)						0.1373		0.1569
Male	275 (78.1)	65 (84.4)	69 (74.2)	86 (81.9)	55 (71.4)		77 (85.6)	
Female	77 (21.9)	12 (15.6)	24 (25.8)	19 (18.1)	22 (28.6)		13 (14.4)	
AECOPD, n/per year	0 (0–1)	1 (0–2)^d^	1 (0–3)^c,d^	0 (0–1)^b^	0 (0–1)^a,b^	< 0.001	0 (0–1)	0.180
CCI, score	4 (3–5)	5 (3–5)^b,d^	4 (3–4)^a,c,d^	4 (4–5)^b,d^	3 (2–3)^a,b,c^	< 0.001	3 (3–4)	0.109
Medication use, n (%)	276 (83.1)	61 (84.7)	79 (91.9)	84 (84.0)	52 (70.3)	0.003	66 (73.3)	0.252
SABA	37 (11.4)	10 (14.3)	15 (17.6)	7 (7.1)	5 (6.9)		10 (11.1)	
LABA	53 (16.3)	13 (18.6)	19 (22.4)	18 (18.4)	3 (4.2)		11 (12.2)	
SAMA	16 (4.9)	4 (5.7)	9 (10.6)	3 (3.1)	0 (0.0)		2 (2.2)	
LAMA	90 (27.7)	26 (37.1)	23 (27.1)	27 (27.6)	14 (19.4)		19 (21.1)	
LABA/LAMA combination	98 (30.2)	18 (25.7)	31 (36.5)	30 (30.6)	19 (26.4)		31 (34.4)	
ICS	38 (11.7)	7 (10.0)	14 (16.5)	11 (11.2)	6 (8.3)		13 (14.4)	
ICS/LABA combination	121 (37.2)	31 (44.3)	32 (37.6)	35 (35.7)	23 (31.9)		30 (33.3)	
LTRA	16 (4.9)	5 (7.2)	3 (3.5)	5 (5.1)	3 (4.2)		4 (4.4)	
Xanthines	27 (8.3)	14 (20.0)	6 (7.1)	3 (3.1)	4 (5.6)		3 (3.3)	
Mucolytics	22 (6.8)	5 (7.1)	9 (10.6)	7 (7.1)	1 (1.4)		8 (8.9)	
NIV, n (%)	45 (12.8)	27 (35.1)	7 (7.5)	10 (9.5)	1 (1.3)	< 0.001	12 (13.5)	1.000
LTOT, n (%)	46 (13.1)	24 (31.2)	19 (20.4)	1 (1.0)	2 (2.6)	< 0.001	13 (14.6)	0.8363
Pulmonary traits								
Lung function								
FEV_1_, % of predicted	55.0 (40.8–70.3)	41 (32–49)^b,c,d^	47 (36–66)^a,c,d^	69 (57–80)^a,b,d^	62 (44–75)^a,b,c^	< 0.001	49.0 (37.0–62.0)	0.075
FVC, % of predicted	80 (66.0–93.8)	64 (53.5–70.0)^b,c,d^	78 (63.0–90.3)^a,c^	93 (80.0–105.5)^a,b,d^	81 (68.5–96.0)^a,c^	< 0.001	78.0 (63.0–91.0)	0.641
FEV_1_/FVC	56.2 (44.1–65.0)	50.3 (41.2–61.9)^c,d^	51.4 (38.7–62.7)^c,d^	59.1 (50–65.1)^a,b^	61.7 (50.2–68)^a,b^	< 0.001	50.0 (42.0–59.2)	0.008
GOLD grades, n (%)						< 0.001		0.196
1	49 (13.9)	0 (0)	7 (7.5)	28 (26.7)	14 (18.2)		10 (11.1)	
2	159 (45.2)	18 (23.4)	39 (41.9)	65 (61.9)	37 (48.1)		34 (37.8)	
3	111 (31.5)	45 (58.4)	34 (36.6)	12 (11.4)	20 (26.0)		34 (37.8)	
4	33 (9.4)	14 (18.2)	13 (14.0)	0 (0)	6 (7.8)		11 (12.2)	
GOLD groups, n (%)						< 0.001		0.003
A	85 (24.1)	7 (9.1)	0 (0)	42 (40.0)	36 (46.8)		22 (24.4)	
B	166 (47.2)	42 (54.5)	51 (54.8)	41 (39.0)	32 (41.6)		45 (50.0)	
C	22 (6.3)	6 (7.8)	1 (1.1)	11 (10.5)	4 (5.2)		4 (4.4)	
D	79 (22.4)	22 (28.6)	41 (44.1)	11 (10.5)	5 (6.5)		18 (20.0)	
Extrapulmonary traits—physical								
BMI, kg/m^2^	26.4 (23.8–29.8)	29.7 (26.2–33.2)^b,d^	24.7 (22.4–27.9)^a,c^	27.7 (25.2–30.5)^b,d^	24.6 (22–27)^a,c^	< 0.001	26.0 (23.9–29.8)	0.540
< 21, n (%)	30 (8.5)	0 (0)	13 (14.0)	3 (2.9)	14 (18.2)	< 0.001	10 (11.1)	0.765
> 30, n (%)	85 (24.1)	35 (45.5)	14 (15.1)	30 (28.6)	6 (7.8)		19 (21.1)	
QMS, kgF	26.2 (20.4–31.6)	25.7 (18.3–30.8)^d^	23.9 (18.2–29.6)^d^	26 (21–30.3)	28.6 (24–33.2)^a,b^	0.002	31.0 (23.9–36.2)	0.000
< 70% of predicted, n (%)	174 (49.4)	48 (62.3)	49 (52.7)	45 (42.9)	32 (41.6)	0.026	15 (16.7)	< 0.001
1minSTS, repetitions	27 (20.8–34.0)	23 (18–27)^c,d^	22 (17–28)^c,d^	28 (23–34)^*^	36 (30–44)^*^	< 0.001	26.5 (22.0–33.0)	0.800
< 70% of predicted, n (%)	133 (37.8)	40 (51.9)	56 (60.2)	24 (23.3)	13 (16.9)	< 0.001	25 (27.8)	0.131
Extrapulmonary traits – symptoms and health status								
CAT, score	14 (8–20)	15 (11–19)^*^	23 (19–27)^*^	9 (6–14)^a,b^	9 (6–13)^a,b^	< 0.001	14.0 (8.0–21.0)	0.702
≥ 10, n (%)	245 (69.6)	64 (83.1)	92 (98.9)	52 (49.5)	37 (48.1)	< 0.001	58 (64.4)	0.347
≥ 18, n (%)	110 (31.3)	23 (29.9)	77 (82.8)	9 (8.6)	1 (1.3)	< 0.001	24 (26.7)	0.399
mMRC, score	2 (1–2)	2 (2–3)^c,d^	3 (2–3)^c,d^	1 (1–2)^a,b^	1 (0–1)^a,b^	< 0.001	1 (1–2)	0.984
≥ 2, n (%)	183 (52.0)	64 (83.1)	81 (87.1)	29 (27.6)	9 (11.7)	< 0.001	43 (47.8)	0.536
HADS, score								
HADS-A	6 (3–9)	5 (3–7)^b^	10 (6–12)^*^	5 (3–7)^b^	5 (3–7)^b^	< 0.001	6.5 (4.2–12.2)	0.243
HADS-A ≥ 8, n (%)	118 (33.5)	18 (23.4)	61 (65.6)	24 (22.9)	15 (19.5)	< 0.001	24 (26.7)	0.591
HADS-D	6 (3–9)	6 (3–9)^b,d^	9 (6–11)^*^	5 (3–8)^b,d^	3 (2–5)^*^	< 0.001	6.0 (3.0–7.0)	0.697
HADS-D ≥ 8, n (%)	125 (35.5)	29 (37.7)	58 (62.4)	30 (28.6)	8 (10.4)	< 0.001	21 (23.3)	0.130
SGRQ, score								
Symptoms	59.3 (± 24.1)	69.4 (± 14.2)^c,d^	76.1 (± 12.6)^c,d^	42.7 (± 20.1)^a,b^	35.3 (± 22.8)^a,b^	< 0.001	48.0 (± 22.2)	0.981
Activities	35.7 (± 21.1)	41.3 (± 14.9)*	51.4 (± 14.9)*	24.6 (± 18.5)*	13.0 (± 10.8)*	< 0.001	55.5 (± 26.2)	0.397
Impact	41.7 (± 19.0)	49.5 (± 12.5)*	61.0 (± 11.3)*	31.2 (± 13.7)*	24.8 (± 12.2)*	< 0.001	31.7 (± 22.4)	0.356
Total	48.1 (± 22.1)	49.9 (± 17.0)^b,c^	63.9 (± 15.6)^*^	30.9 (± 19.4)^a,b^	37.0 (± 20.5)^b^	< 0.001	41.5 (± 21.6)	0.953
Total ≥ 25, n (%)	271 (77.0)	75 (97.4)	93 (100)	69 (65.7)	34 (44.2)	< 0.001	71 (78.9)	0.695
Total ≥ 46, n (%)	153 (43.5)	45 (58.4)	86 (92.5)	18 (17.1)	4 (5.2)	< 0.001	34 (37.8)	0.412
Extrapulmonary traits—behavioural								
Smoking status, n (%)						< 0.001		0.192
Current	52 (14.8)	6 (7.8)	13 (14.0)	9 (8.6)	24 (31.2)		12 (13.3)	
Former	221 (62.8)	52 (67.5)	57 (61.3)	66 (62.9)	46 (59.7)		65 (72.2)	
Never	79 (22.4)	19 (24.7)	23 (24.7)	30 (28.6)	7 (9.1)		13 (14.4)	
Pack-years	32.3 (2.9–60)	48 (0.1–87.5)	28 (2–52)	30 (0–52.5)	30 (15–45)	0.112	50.0 (30.0–90.0)	< 0.001
BPAAT, score	1 (0–4)	0 (0–4)^d^	0 (0–4)	1 (0–4)	2 (0–4)^a^	0.003	1 (0.5–4)	< 0.001
Insufficiently active, n (%)	242 (69.1)	54 (70.1)	68 (73.9)	74 (71.2)	46 (59.7)	0.221	61 (68.5)	0.912
Sufficiently active, n (%)	108 (30.9)	23 (29.9)	24 (26.1)	30 (28.8)	31 (40.3)		28 (31.5)	

Continuous variables are expressed as mean (± standard deviation) or median [first quartile—third quartile] according to their distribution. Categorical variables were expressed as absolute frequency (%)

*AECOPD* acute exacerbations of COPD, *BMI*, body mass index, *BPAAT* Brief Physical Activity Assessment Tool, *CCI* Charlson Comorbidity Index, *CAT* COPD Assessment Test, *FEV*_*1*_ forced expiratory volume in 1 s, *FVC* forced vital capacity, *GOLD* Global Initiative for Chronic Obstructive Lung Disease; HADS, The Hospital Anxiety and Depression Scale, *LTOT* Long-Term Oxygen Therapy, *SABA* Short-Acting Beta Agonists, *SAMA* Short-acting muscarinic-antagonist; *LABA* Long-acting beta-agonists, *LAMA* Long-acting muscarinic antagonists, *ICS* Inhaled corticosteroids, *LTRA* Leukotriene receptor antagonist, *mMRC* Modified British Medical Research Council questionnaire, *NIV* Non-Invasive Ventilation, *QMS* Quadriceps muscle strength, *SGRQ* Saint George’s Respiratory Questionnaire; 1minSTS, 1-min Sit-to-Stand Test

*p < 0.05 when compared with all other profiles

^a^p < 0.05 vs Profile 1

^b^p < 0.05 vs Profile 2

^c^p < 0.05 vs Profile 3

^d^p < 0.05 vs Profile 4

Profiles 1 and 2 were mostly composed of people with severe airflow obstruction, medium–high levels of dyspnoea, medium–high impact of the disease and low lower-limb muscle function, functional status and health-related quality of life. Profile 2 integrated the higher proportion of people on long-term oxygen therapy, with anxiety and depression symptoms, highest impact of the disease and poorest health-related quality of life.

Profiles 3 and 4 included mostly people with moderate airflow obstruction, less dyspnoeic, with low impact of the disease, high lower-limb muscle function and functional status, and better health-related quality of life. Profile 4 also presented the highest proportion of sufficiently active people. Further details are presented in Additional file 1: Figure S3.

### Decision tree

**Fig. 3:**
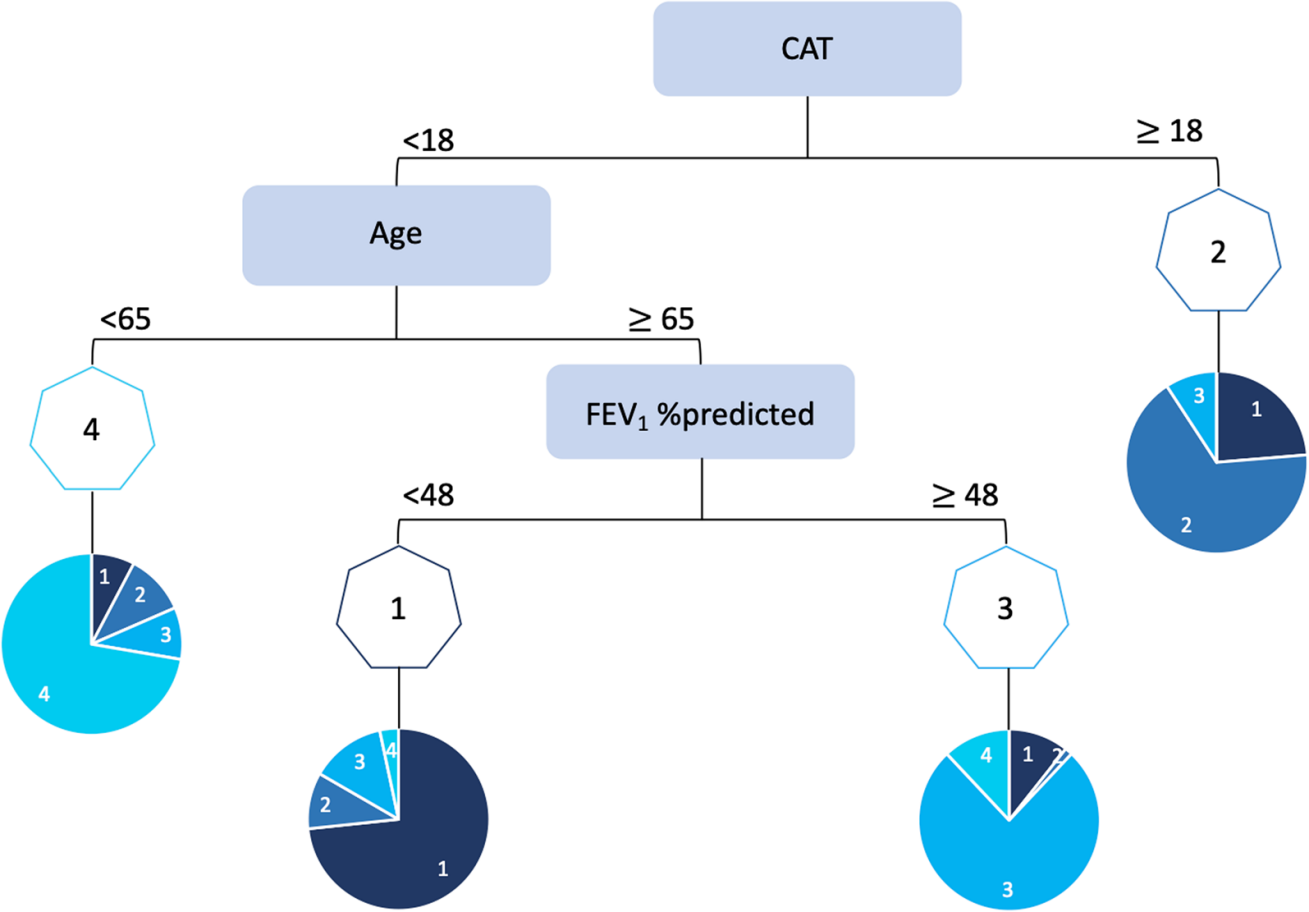
Decision tree to assign people with chronic obstructive pulmonary disease (COPD) to the identified profiles (1, 2, 3 and 4). Cut-off points were 18 for the COPD Assessment Test (CAT), 65 years for age and 48% of predicted for the forced expiratory volume in 1 s (FEV_1_). Pie charts represent the proportion of people with COPD correctly assigned to each profile using the decision tree (using the actual profile provided in Fig. 2 as the criterion)

The Random Forest identified CAT (cut-off 18 points), age (cut-off 65 years), and FEV_1_% predicted (cut-off 48% predicted) as the most informative variables for the decision tree (Fig. 3) of the four profiles (Additional file). The tree assigned correctly 71.7% of people with COPD to their actual profile, thus a substantial agreement (Cohen’s Kappa = 0.62, p < 0.001) was observed.

### Stability of the profiles

**Fig. 4:**
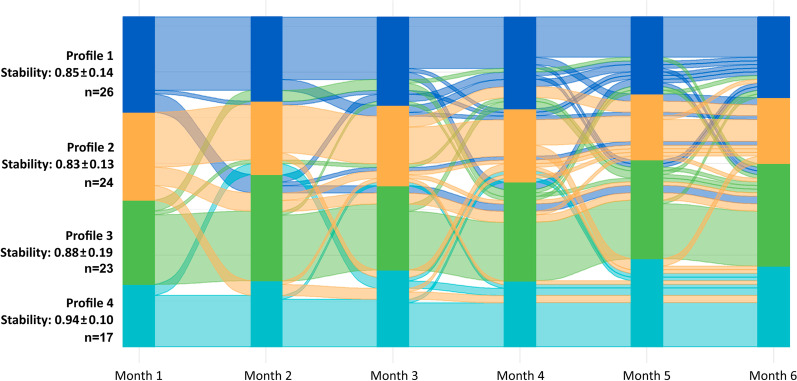
Flow of people with chronic obstructive pulmonary disease (n = 90) across profiles (1, 2, 3 and 4) during the six-month follow-up period

From the 90 participants followed-up, 26 (28.9%) were in profile 1, 24 (26.7%) in profile 2, 23 (25.5%) in profile 3 and 17 (18.9%) in profile 4 (Additional file 1: Table S1). 42% (11/26) of people in profile 1, 25% (6/24) in profile 2, 65% (15/23) in profile 3 and 71% (12/17) in profile 4 remained in the same profile over time. Therefore, profile 4 presented the higher stability score (0.94 ± 0.10), followed by profile 3 (0.88 ± 0.19), profile 1 (0.85 ± 0.14) and profile 2 (0.83 ± 0.13). Overall, 48.9% (n = 44) of people remained in the same profile during the six months (stability score = 1). The other participants (n = 43; 47.8%) remained predominantly in one of the profiles (i.e., for at least 3 months) but moved between two profiles. Three participants (3.3%) moved across three profiles over time. The overall percentage of stability was 86.8 ± 15%. Figure 4 shows the flow of participants across profiles during the follow-up.

## Discussion

Four distinct profiles and respective treatable traits of people with COPD were identified and described using simple and widely available measures. Heterogeneity within each profile and overlap in some treatable traits existed. We proposed a simple decision tree (CAT < 18 or ≥ 18 points; age < 65 or ≥ 65 years; FEV_1_ < 48 or ≥ 48% predicted) that can aid healthcare professionals in clinical practice to allocate each person to a specific profile quickly, even with minimal resources. This is of most importance, as it can rapidly guide personalised assessments and decision-making regarding the best treatments for each treatable trait. We also showed that 51.1% of the sample changed their profile over a six-month period and that those more symptomatic, with moderate-high impact of the disease and lower limb muscle function, functional status and health-related quality of life (profiles 1 and 2) were the ones less stable in their profile. Our findings suggest that frequent assessments of people with COPD might be needed to ensure that the most adequate personalised interventions are offered.

Despite the enormous variability of published clinical phenotypes/profiles in COPD [3–12, 32], some common ground to our study seems to exist, i.e., profiles composed of older, overweight and dyspnoeic people and others with younger people with nutritional depletion [5–7, 11] and high as well as low impact of the disease, lower-limb muscle function, functional status and health-related quality of life may be present in people with similar airflow limitation [5, 11, 12]. Thus, our findings reinforce the previously acknowledged need to include comprehensive assessments in people with COPD, that go beyond pulmonary measures [9, 12, 15, 33]. Such assessments, even if applied with minimal resources, as demonstrated in our study, allow the easy identification of extrapulmonary treatable traits within each profile, which is of most importance for the daily management of COPD since they can be modified with person-centred interventions [14].

In our study, we were able to identify that people with COPD from profiles 1, 2 and 3 might benefit from pulmonary rehabilitation (exercise, education and psychosocial support) to target their activity-related dyspnoea, impact of disease, lower-limb muscle dysfunction and/or functional status impairment [34]. Profiles among people with COPD who could benefit from pulmonary rehabilitation and physical activity promotion to address their symptoms, exercise intolerance and functional status impairments have been previously reported [8, 10, 32, 35–37]. Those in profiles 1 and 3 might also benefit from dietary counselling and calorie restriction plus resistance exercise training [38, 39] and referral for comorbidities’ management [40], due to their high BMI and severity of comorbidities. Indeed, different body composition and comorbidity profiles which involve several body systems in people with COPD have been acknowledged and multidisciplinary care approaches advocated [7, 41]. People in profile 2 showed the worst emotional status. This was not surprising, as a specific emotionally dysfunctional cluster has been previously identified in people with COPD [9]. People in profile 2 might benefit from a specific psychological assessment and possibly cognitive behavioural therapy [42, 43]. Lastly, people in profile 4 exhibit much better health status than those in the other profiles, indicating that physical activity counselling [44] and self-management programmes [45] might be the most appropriate interventions as well as nutritional support to manage their underweight [46]. In fact, regular physical activities in the local community have been recommended for older people with COPD who integrate the profile of no or low disease burden and better health status [10]. This recommendation is based on the rationale that any additional increase in physical activity and decrease in sedentary behaviour is important to improve health outcomes in this population [47, 48].

The proposed and validated simple decision tree facilitates the identification of these profiles in clinical practice. Similar cut-offs have been previously proposed: for CAT when referring to exercise-based interventions[10] and to better differentiate symptom burden in COPD [2]; as well as for age [5] and for FEV_1_% predicted when predicting mortality [49], which suggests that these cut-offs might be the most adequate to differentiate health status of people with COPD. The quick allocation of each person to a profile can aid decision-making regarding prioritisation of assessments and identification of specific targets to improve patient-related outcomes [10], saving time and financial resources spent in first assessments. However, it should never preclude healthcare professionals from conducting individual comprehensive assessments [9, 12].

Our work also emphasised the need for regular assessments as we found that 51.1% of people with COPD assigned to one profile changed to another over time, and hence some of their treatable traits might have changed too. Migration across profiles within one [49] or two [50] years has been previously observed, mainly driven by changes in symptoms (CAT), lung function (FEV_1_), functional status (Six-minute walking test) [50] and physical activity levels [49]. Our findings show that this variability was especially evident in the more “deteriorated” profiles, mainly due to changes in the CAT score, and confirms that not all patients progress unfavourably [49, 50]. It is already known that dramatic changes can occur over time in variables that capture patients’ real life, such as symptoms (e.g., fatigue) and functional status [51], whilst FEV_1_ remains relatively stable [52]. In fact, the profile of patients with COPD with prolonged hospitalizations has been found not to be associated with disease severity assessed by lung function [53]. Therefore, identifying and tackling extrapulmonary treatable traits as early as possible seems fundamental to optimise outcomes in COPD, independently of the resources available. Reasons causing the shift among our profiles (e.g., change in lifestyle, acute exacerbation occurrence) would be of interest but it was beyond the scope of our study. The literature on the variability/stability of profiles and respective treatable traits over time is yet scarce and further investigation is warranted.

Strengths of our study include the integration of people with COPD from all GOLD grades and groups, living in the community, being a good representation of their daily lives. Our findings are prevenient from comprehensive assessments based on simple and widely available measures, and a simple decision tree to identify each profile and respective treatable traits is provided. Variability of each profile was evaluated over time, showing the need of personalising assessments and their frequency.

Limitations of this study include the relatively small number of participants included and proportion of females in our sample. All efforts were conducted to avoid missing data. To minimise participants’ transportation and discomfort, data were mostly collected during their routine appointments with their clinician (at the hospital or primary healthcare centre), where time, space and equipment constraints exist. This decision had, however, implications in our study as often participants were not available to complete the full assessments. Conducting the assessment in a separate occasion of the regular appointment and/or agreeing to collect data in two different moments, could minimise this constraint. Nevertheless, data gathered in this work represents approximately 5% of the COPD population in our country and were collected across different settings and geographic regions, representing real-world data. Similar samples with unbalance proportion of female/male have been widely reported in COPD profiles [5–7, 11] however, we acknowledge that this may limit the findings to all people with COPD. Our follow-up period was also relatively short, limiting our understanding about the impact of profile migration in the overall disease development and important outcomes (e.g., risk of exacerbations). Longer longitudinal studies, with the inclusion of a higher proportion of females, and exploring associations with clinically relevant variables are warranted to confirm and further develop our findings. We did not include people who dropped out from follow-up as we wanted to assess stability of the profiles and respective treatable traits, thus we are unable to infer if the profile of those lost to follow-up differed from those included. Although we performed a comprehensive assessment, many other important measures could be considered when profiling and identifying treatable traits in people with COPD (e.g., fatigue impairment, sleep disturbances, sedentary behaviour)[14]. Core treatable traits in COPD and efficacy of targeting treatments to them are yet unknown but should be addressed in future investigations.

## Conclusion

Four profiles and their treatable traits were identified in people with COPD using minimal resources. A simple decision tree (CAT < 18 or ≥ 18 points; age < 65 or ≥ 65 years; FEV_1_ < 48 or ≥ 48% predicted) is now available to facilitate routine allocation of people to these profiles. Heterogeneity within each profile and overlap in treatable traits existed, as well as migration across profiles (51.1% of the sample) over time, especially in those more symptomatic and with worse functional status. Our findings provide additional evidence emphasising the need of frequent personalised assessments to identify treatable traits and ensure the most adequate person-centred intervention is offered to people with COPD. Nevertheless, future research namely on the validation of our decision tree and profiles in other samples would be beneficial.

## Take home message

Profiles and treatable traits in COPD can be identified even with minimal resources. People with COPD may change profile and treatable traits over time hence, regular assessments to deliver goal-targeted personalised treatments are needed.

## Supplementary Information

**Additional file 1.** An additional file provides more details on the statistical analysis performed and further information on the results for GAP statistics, sample characteristics and distribution.

## Data Availability

The database used to produce this paper will not be shared because further analyses are still being conducted. Nevertheless, additional information on the data will be provided upon request to the authors.

## Abbreviations

1minSTS: 1-Min sit-to-stand test
BMI: Body mass index
BPAAT: Brief physical activity assessment tool
CAT: COPD assessment test
CCI: Charlson comorbidity index
COPD: Chronic obstructive pulmonary disease
FEV_1_: Forced expiratory volume in one second
GOLD: Global initiative for chronic obstructive lung disease
HADS: Hospital anxiety and depression scale
HADS-A: Hospital anxiety and depression scale—anxiety subscale
HADS-D: Hospital anxiety and depression scale—depression subscale
mMRC: Modified British Medical Research Council dyspnoea questionnaire
QMS: Quadriceps muscle strength
SGRQ: Saint George’s respiratory questionnaire
