# Supplementary material for: COPD profiles and treatable traits using minimal resources: identification, decision tree and stability over time
Source: Respir Res. 2022 Feb 14;23:30. doi: 10.1186/s12931-022-01954-6 (PMC8842856; doi:10.1186/s12931-022-01954-6)
Supplement: Supplementary file 1 — Additional file 1. An additional file provides more details on the statistical analysis performed and further information on the results for GAP statistics, sample characteristics and distribution. [file 12931_2022_1954_MOESM1_ESM.docx]

# additional file 1

**METHODS**

***Statistical analysis***

All analyses were performed in R (version 3.6.1) with a level of significance set at p<0.05. Descriptive statistics were used to characterise the sample. Normality of data distribution was tested with the Shapiro-Wilk test. Continuous variables were expressed as mean±standard deviation or median [first quartile; third quartile]. Categorical variables were expressed as absolute and/or relative frequency. Comparison between participants’ characteristics and between the total and follow-up sample were analysed using t-test or Mann Whitney U-test, for continuous variables; and chi-squared or Fisher’s test, for categorical variables. Differences among profile characteristics were analysed using univariate ANOVA followed by Tukey’s multiple comparison test or Kruskal–Wallis test followed by Dunn’s multiple comparison test, for continuous variables; and chi-squared or Fisher’s test, for categorical variables, according to their assumptions.

***Establishment of profiles***

k-medoid is a classical partitioning technique of clustering which divides the data set into k clusters, with the number k of clusters assumed known a priori(1). K-medoids is related to k-means and is commonly used in domains that require robustness to outlier data. The goal of both methods is to divide a set of measurements into clusters so that the subsets minimize the sum of distances between a measurement and the center of the cluster. In the k-means algorithm, the centre of the subset is the mean of measurements in the subset; while in the k-medoids algorithm, the centre of the subset is a member of the subset. A medoid is less influenced by outliers or other extreme values than a mean. Moreover, k-medoids is more robust to noise and outliers, as compared to k-means, because it minimizes a sum of pairwise dissimilarities instead of a sum of squared Euclidean distances (1).

The result of the partition algorithm depends on the number of clusters k, which is often hard to choose a priori. Therefore, it is common practice to run the method for several values of k, and then select the “best” value of k as the one which optimizes a certain criterion called a validity index. We used the GAP statistic to identify the optimal number of non-random clusters (2). For each number of clusters k, the Gap statistic compares log(W_k_) with its expectation, where W_k_ is the within-cluster dispersion and the latter is defined via bootstrapping. The idea is to compute a stability score not only on the actual data set, but also on “null data sets” drawn from a null reference distribution (a uniform distribution on the hypercube determined by the ranges of the data). The optimal number of clusters is the one that maximizes the gap statistic. We have examined different values of k, between 2 and 10, considering 500 simulated random null data sets to each case.

When variables used in the generation of clusters are strongly correlated, some features get a higher weight than others. Using uncorrelated features ensures that strongly correlated variables do not dominate the cluster assignment. Therefore, we applied Principal Component Analysis on the set of selected variables obtaining a set of linearly uncorrelated variables (the principal components). The number of components to retain was selected such that at least 80% of the variance was explained. Scores associated to those first principal components yield a data matrix, on which the clustering algorithm, k-medoids, was applied.

***Decision tree***

A decision tree analysis is a supervised learning method that continuously split data according to a certain parameter and a binary decision. It was developed to facilitate the allocation of each person to their specific profile. Variable selection was performed to identify the most reliable subset of variables for the construction of a decision tree from the 13 variables used in the clustering procedure (Age, BMI, Pack-years, CCI, FEV_1_ percentage predicted, FVC percentage predicted, CAT, mMRC, HADS-A, HADS-D, SGRQ, QMS and 1minSTS). Random Forest was used to rank the importance of variables. The top variables were selected for the decision tree construction. In the presence of highly correlated variables, one is selected and the other discarded.

Random Forest is an ensemble of random decision trees, each one built from a different sample of the original data. The predictions of all random trees are combined and the algorithm chooses the classification having the most votes as its prediction result(3). This technique also produces an identification of variables that are important in a great number of decision trees, which provides suggestions in terms of variable selection. The prediction accuracy is measured after randomly permuting the variable values, keeping all other variables the same, to determine the importance of a specific variable. Then, the difference in prediction accuracy on the initial and the permuted data is measured. The importance score for the variable, named Mean Decrease Accuracy, is computed by averaging the decrease in accuracy across all trees(3). Features which produce large values for this score are more important for classification of the data than features which produce small values.

The entire dataset was split into training and validation datasets to establish the thresholds for each identified variable in the decision tree. These threshold values ​​may fluctuate according to the training dataset. Thus, the procedure of split the entire dataset into training and validation datasets (to create a decision tree based on the first one and to validate it in the second) was repeated 100 times. The final proposed decision tree was the one that maximised the overall accuracy.

**RESULTS**

***Number of profiles***

The maximum of gap statistics was obtained for the four-cluster solution (Figure S1), which suggest the existence of four profiles presented in the data.

**Figure S1.** Gap statistic value by number of clusters. The maximum value (optimal number) is reached with four clusters suggesting that four different profiles exist among the cohort of people with chronic obstructive pulmonary disease (COPD) (n=352).

Each profile included people with COPD from all grades of airflow obstruction limitation (Figure S2 a) and GOLD groups (Figure S2 b).


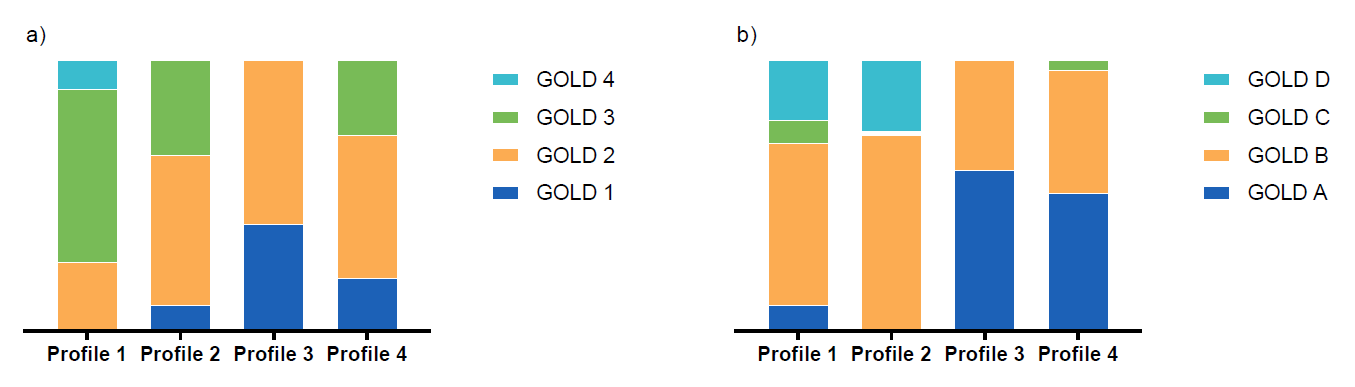


**Figure S2.** Sample distribution in each profile (1, 2, 3 and 4) according to a) airflow limitation GOLD grades (1, 2, 3, and 4) and b) GOLD groups (A, B, C, D).

Detailed characteristics of each profile of people with COPD per variable collected are presented in Figure S3.


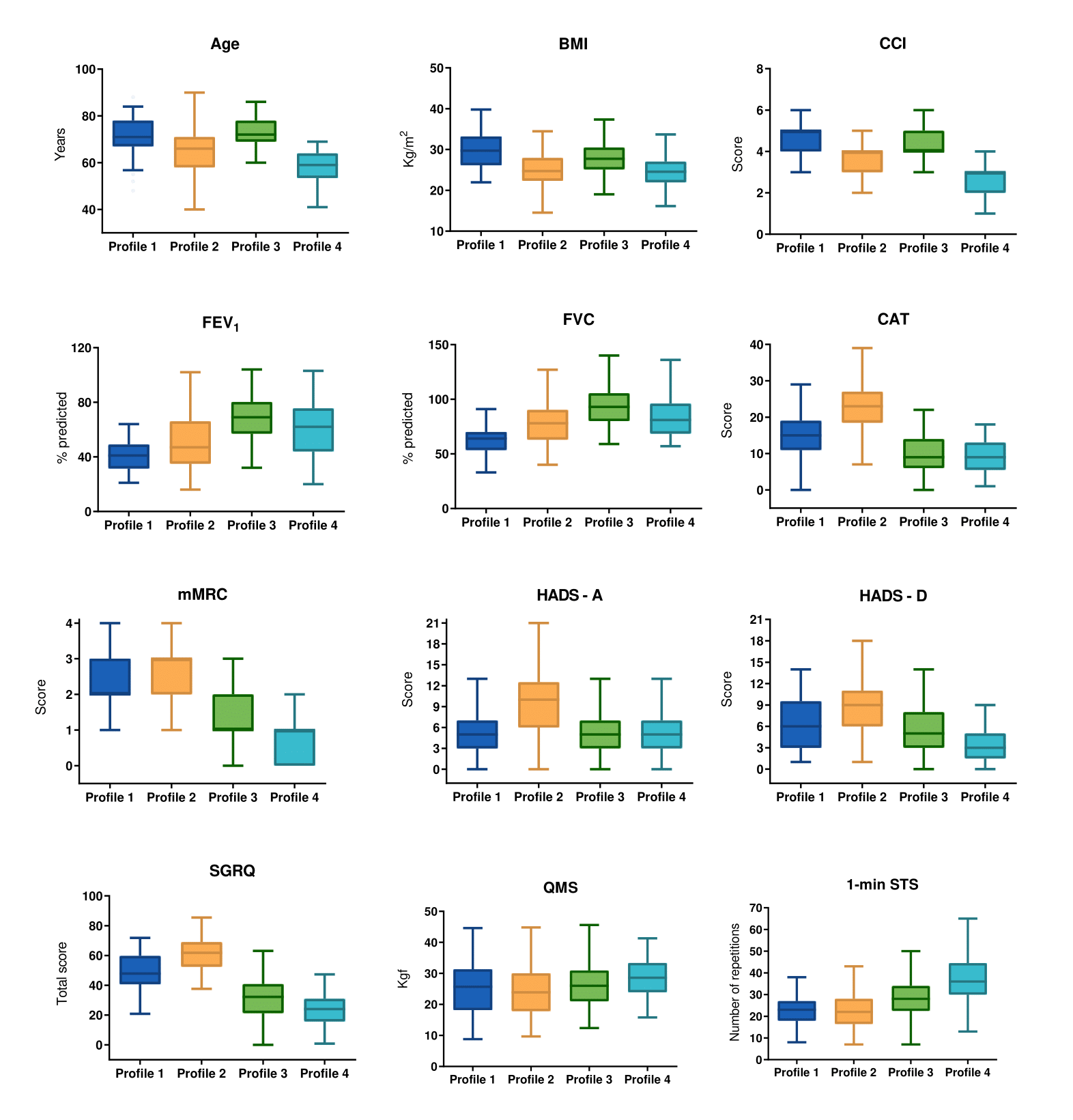
**Figure S3:** Characteristics of people with chronic obstructive pulmonary disease (COPD), in each of the four profiles identified by the k-medoids partitioning algorithm (n=352), for each variable explored. The thick central horizontal line within each box plot represents the median, the box represents the interquartile range, and the vertical lines the range from the minimum to the maximum value of each cluster of patients for each variable: age (years), BMI, Body Mass Index (kg/m^2^); CCI, Charlson Comorbidity Index (points); FEV_1_, Forced Expiratory Volume in one second percentage predicted; FVC, Forced Vital Capacity percentage predicted; CAT, COPD Assessment Test total score (points); mMRC, modified Medical Research Council Questionnaire (points); HADS-A, The Hospital Anxiety and Depression Scale – Anxiety (points); HADS-D, The Hospital Anxiety and Depression Scale – Depression (points); SGRQ, The Saint George’s Respiratory Questionnaire (points); QMS, Quadriceps isometric muscle strength (KgF); 1-min STS, 1-minute sit-to-stand test (repetitions) per profile.

***Decision tree***

Random Forest was used to rank the variables’ importance for the classification in the four clusters. Mean decrease accuracy values were: SGRQ (0.057), CAT (0.051), Age (0.050), CCI (0.050), mMRC (0.047), FVC% predicted (0.034), FEV_1_% predicted (0.027), BMI (0.022), HADS-A (0.017), 1minSTS (0.015), HADS-D (0.015), Packyears (0.006), QMS (0.003). Several subsets of strongly correlated variables were found in the top positions random. Therefore, CAT, Age and FEV_1_% predicted were the three variables identified as being the most important for the classification of participants in the four profiles: SGRQ was discarded as it was not present monthly in the longitudinal dataset; Age and CCI were highly correlated (spearman rho =0.81, p<0.001), therefore only Age was considered due to its simplicity to be used in clinical practice; CAT and mMRC were strongly correlated (spearman rho =0.56, p<0.001), CAT had a slightly higher accuracy and therefore was chosen; FEV_1_% predicted and FVC% predicted were strongly correlated (spearman rho =0.56, p<0.001), FEV_1_% predicted was chosen as it is commonly used to stratify severity of airflow obstruction.

The proposed decision tree was found to be highly stable, with only minor fluctuations in the threshold values of each variable (mean value of correct predictions of the 100 decision trees was 69%).

***Stability of the profiles over time***

Of the 90 people with COPD that were followed-up during six months, 26 (28.9%) were in profile 1, 24 (26.7%) in profile 2, 23 (25.5%) in profile 3 and 17 (18.9%) in profile 4. Detailed characteristic are presented in Table S1.

**Table S1.** Characteristics of people with chronic obstructive pulmonary disease (COPD) included in the longitudinal subsample (n=90) and for each of the four profiles.

|  | Longitudinal subsample (n=90) | | Profile 1  (n=26) | Profile 2  (n=24) | | | Profile 3  (n=23) | Profile 4  (n=17) | | p-value  (comparisons across profiles) | |
| --- | --- | --- | --- | --- | --- | --- | --- | --- | --- | --- | --- |
| Age, years | 68 (62-72) | | 71^b,d^  (68-76) | 66^c,d^(59-69) | | | 70^b,d^(68-75) | 58^*^ (54-61) | | <0.001 | |
| Sex, n (%) |  | |  |  | | |  |  | |  | |
| Male | 77 (85.6) | | 25 (96.2) | 17 (70.8) | | | 20 (87.0) | 15 (88.2) | | 0.080 | |
| Female | 13 (14.4) | | 1 (3.8) | 7 (29.2) | | | 3 (13.0) | 2 (11.8) | |  |  |
| AECOPD, n/per year | 0 (0-1) | | 0 (0-1) | 1^c^ (0-3) | | | 0^b^ (0-0) | 0 (0-1) | | 0.022 | |
| CCI, score | 3 (3-4) | | 2^b,d^ (2-3) | 2^a^ (1-2) | | | 2^d^ (2-3) | 1^*^ (1-2) | | <0.001 | |
| Medication use, n (%) | 66 (73.3) | | 18 (69.2) | 17 (70.8) | | | 16 (69.6) | 15 (88.2) | | 0.726 | |
| SABA | 10 (11.1) | | 6 (23.1) | 3 (12.5) | | | 1 (4.3) | 0 (0.0) | | 0.017 | |
| LABA | 11 (12.2) | | 4 (15.4) | 6 (25.0) | | | 0 (0.0) | 1 (5.9) | | 0.009 | |
| SAMA | 2 (2.2) | | 1 (3.8) | 1 (4.2) | | | 0 (0.0) | 0 (0.0) | | 0.436 | |
| LAMA | 19 (21.1) | | 6 (23.1) | 6 (25.0) | | | 3 (13.0) | 4 (23.5) | | 0.644 | |
| LABA/LAMA combination | 31 (34.4) | | 7 (26.9) | 7 (29.2) | | | 10 (43.5) | 7 (41.2) | | 0.596 | |
| ICS | 13 (14.4) | | 4 (15.4) | 5 (20.8) | | | 3 (13.0) | 1 (5.9) | | 0.314 | |
| ICS/LABA combination | 30 (33.3) | | 8 (30.8) | 8 (33.3) | | | 6 (26.1) | 8 (47.1) | | 0.891 | |
| LTRA | 4 (4.4) | | 2 (7.7) | 1 (4.2) | | | 0 (0.0) | 1 (5.9) | | 0.445 | |
| Xanthines | 3 (3.3) | | 2 (7.7) | 0 (0.0) | | | 0 (0.0) | 1 (5.9) | | 0.223 | |
| Mucolytics | 8 (8.9) | | 2 (7.7) | 1 (4.2) | | | 4 (17.4) | 1 (5.9) | | 0.365 | |
| NIV, n (%) | 12 (13.5) | | 5 (19.2) | 1 (4.2) | | | 5 (21.7) | 1 (5.9) | | 0.208 | |
| LTOT, n (%) | 13 (14.6) | | 9 (34.6) | 3 (12.5) | | | 1 (4.3) | 0 (0.0) | | 0.007 | |
| ***Pulmonary traits*** |  | |  |  | | |  |  | |  | |
| Lung function |  | |  |  | | |  |  | |  | |
| FEV_1_, % of predicted | 49.0 (37.0-62.0) | | 37.5^c,d^ (31.0-43) | 44.0 (33.5-67.0) | | | 60.0^a^ (51.7-65.0) | 66^a^ (49.0-74.0) | | <0.001 | |
| FVC, % of predicted | 78.0 (63.0-91.0) | | 71.0^c,d^ (60.0-79.0) | 74.5 (61.5-92.0) | | | 86^a^ (75.3-97.0) | 83.0^a^ (78.0-101.0) | | 0.008 | |
| FEV_1_/FVC | 50.0 (42.0-59.2) | | 41.0^*^ (33.2-48.0) | 53.0^a^ (42.5-58.0) | | | 57.8^a^ (52.0-60.5) | 58.0^a^ (47.0-68.1) | | <0.001 | |
| GOLD grades, n (%) |  | |  |  | | |  |  | |  | |
| 1 | 10 (11.1) | | 0 (0.0) | 3 (13.1) | | | 2 (8.7) | 5 (29.4) | | <0.001 | |
| 2 | 34 (37.8) | | 3 (11.6) | 7 (30.4) | | | 18 (78.3) | 6 (35.3) | |  |  |
| 3 | 34 (37.8) | | 18 (69.2) | 9 (39.1) | | | 3 (13.0) | 4 (23.5) | |  |  |
| 4 | 11 (12.2) | | 5 (19.2) | 4 (17.4) | | | 0 (0.0) | 2 (11.8) | |  |  |
| GOLD groups, n (%) |  | |  |  | | |  |  | |  | |
| A | 22 (24.4) | | 7 (26.9) | 1 (4.3) | | | 6 (26.1) | 8 (47.1) | | 0.032 | |
| B | 45 (50.0) | | 15 (57.7) | 12 (52.2) | | | 12 (52.2) | 6 (35.3) | |  |  |
| C | 4 (4.4) | | 1 (3.9) | 0 (0.0) | | | 2 (8.7) | 1 (5.9) | |  |  |
| D | 18 (20.0) | | 3 (11.5) | 10 (43.5) | | | 3 (13.0) | 2 (11.7) | |  |  |
| ***Extrapulmonary traits - physical*** | | | | | | | | | | | |
| BMI, kg/m^2^ | 26.0 (23.9-29.8) | | 24.9 (23.4-28.3) | 25.3 (22.8-30.0) | | | 27.9 (25.0-29.6) | 25.9 (23.9-28.5) | | 0.438 | |
| < 21, n (%) | 10 (11.1) | | 2 (7.7) | 5 (20.8) | | | 2 (9.1) | 1 (6.3) | | 0.622 | |
| > 30, n (%) | 19 (21.1) | | 6 (23.1) | 6 (25.0) | | | 5 (22.7) | 2 (12.5) | |  |  |
| QMS, kgF | | 31.0 (23.9-36.2) | 30.6 (26.3-36.5) | | 26.1^d^ (20.6-33.8) | 31.0^d^ (21.2-35.4) | | 35.4^b,c^ (30.9-38.0) | 0.027 | |  |
| < 70 % of predicted, n (%) | | 15 (16.7) | 2 (7.7) | | 6 (25.0) | 5 (21.7) | | 2 (12.5) | 0.321 | |  |
| 1minSTS, repetitions | | 26.5 (22.0-33.0) | 26.5 (22.0-30.0) | | 24.5 (18.5-32.5) | 27.0 (23.0-30.0) | | 33.0 (23.0-41.5) | 0.100 | |  |
| < 70 % of predicted, n (%) | | 25 (27.8) | 7 (26.9) | | 11 (47.8) | 5 (22.7) | | 2 (13.3) | 0.123 | |  |
| ***Extrapulmonary traits – symptoms and health status*** | | | | | | | | | | |  |
| CAT, score | | 14.0 (8.0-21.0) | 14.5^b^ (8.0-21.0) | | 21.0^*^ (16.0-28.5) | 12.0^b^ (7.0-15.0) | | 8.5^b^ (5.0-12.5) | <0.001 | |  |
| ≥ 10, n (%) | | 58 (64.4) | 13 (50.0) | | 24 (100) | 13 (56.5) | | 8 (47.1) | <0.001 | |  |
| ≥ 18, n (%) | | 24 (26.7) | 0 (0.0) | | 24 (100) | 0 (0.0) | | 0 (0.0) | <0.001 | | |
| mMRC, score | | 1 (1-2) | 2^c,d^ (1-3) | | 2^c,d^ (1-3) | 1^a,b^ (1-2) | | 1^a,b^ (0.5-1) | <0.001 | |  |
| ≥ 2, n (%) | | 43 (47.8) | 17 (65.4) | | 17 (70.8) | 7 (30.4) | | 2 (12.5) | <0.001 | |  |
| HADS, score | |  |  | |  |  | |  |  | |  |
| HADS-A | | 6.5 (4.2-12.2) | 4.0 (2.0-7.5) | | 9.0 (5.0-12.0) | 5.0 (3.5-6.0) | | 4.0 (4.0-6.0) | 0.466 | |  |
| HADS-A ≥ 8, n (%) | | 24 (26.7) | 6 (32.1) | | 14 (58.3) | 4 (17.4) | | 0 (0.0) | <0.001 | |  |
| HADS-D | | 6.0 (3.0-7.0) | 5.5 (3.5-7.0) | | 7.0 (6.0-11.0) | 5.0 (3.0-6.5) | | 3.0 (2.0-5.5) | 0.544 | |  |
| HADS-D ≥ 8, n (%) | | 21 (23.3) | 5 (19.2) | | 10 (41.7) | 4 (17.4) | | 2 (11.8) | 0.089 | |  |
| SGRQ, score | |  |  | |  |  | |  |  | |  |
| Symptoms | | 48.0 (±22.2) | 44.5^b^ (±20.9) | | 60.9^*^ (±13.8) | 36.0^b^ (±20.5) | | 43.1^b^ (±17.7) | <0.001 | |  |
| Activities | | 55.5 (±26.2) | 62.8^c,d^ (±19.2) | | 69.5^c,d^ (±20.8) | 46.0^a,b^ (±20.1) | | 35.5^a,b^ (±23.3) | <0.001 | |  |
| Impact | | 31.7 (±22.4) | 25.0^*^ (±16.6) | | 47.0^*^ (±19.3) | 21.8^a,b^ (±16.5) | | 16.8^a,b^ (±14.5) | <0.001 | |  |
| Total | | 41.5 (±21.6) | 44.8^*^ (±15..0) | | 56.1^*^ (±16.6) | 31.6 ^a,b^ (±15.2) | | 26.6 ^a,b^ (±16.3) | <0.001 | |  |
| Total ≥ 25, n (%) | | 71 (78.9) | 25 (96.2) | | 2 (8.3) | 15 (65.2) | | 9 (52.9) | <0.001 | |  |
| Total ≥ 46, n (%) | | 34 (37.8) | 12 (46.2) | | 17 (70.8) | 3 (13.0) | | 2 (11.8) | <0.001 | | |
| **Extrapulmonary traits - behavioural** | | |  | |  |  | |  |  | | |
| Smoking status, n (%) |  | |  |  | | |  |  | |  | |
| Current | 12 (13.3) | | 2 (7.7) | 4 (16.7) | | | 2 (8.7) | 4 (23.5) | | 0.173 | |
| Former | 65 (72.2) | | 19 (73.1) | 14 (58.3) | | | 20 (87.0) | 12 (70.6) | |  |  |
| Never | 13 (14.4) | | 5 (19.2) | 6 (25.0) | | | 1 (4.3) | 1 (5.9) | |  |  |
| Pack-years | 50.0 (30.0-90.0) | | 69.8 (42.5-111.5) | 53.0 (22.0-150.0) | | | 51.0 (30.0-75.0) | 38.0 (18.8-50.0) | | 0.116 | |
| BPAAT, score | | 1 (0.5-4) | 1 (1-4) | | 1 (0-4) | 1 (1-4) | | 1 (0-4) | 0.440 | | |
| Insufficiently active, n (%) | | 61 (68.5) | 19 (73.1) | | 16 (66.7) | 16 (69.6) | | 10 (62.5) | 0.903 | | |
| Sufficiently active, n (%) | | 28 (31.5) | 7 (26.9) | | 8 (33.3) | 7 (30.4) | | 6 (37.5) |  |  |  |

Legend: Continuous variables are expressed as mean (±standard deviation) or median [first quartile; third quartile] according to their distribution. Categorical variables were expressed as absolute frequency (%).

AECOPD, acute exacerbations of COPD; BMI, body mass index; CCI, Charlson Comorbidity Index; CAT, COPD Assessment Test; FEV_1_, forced expiratory volume in 1 second; FVC, forced vital capacity; GOLD, Global Initiative for Chronic Obstructive Lung Disease; HADS, The Hospital Anxiety and Depression Scale; LTOT, Long-Term Oxygen Therapy; SABA, Short-Acting Beta Agonists; SAMA, Short-acting muscarinic-antagonist; LABA, Long-acting beta-agonists; LAMA, Long-acting muscarinic antagonists; ICS, Inhaled corticosteroids; LTRA, Leukotriene receptor antagonist; mMRC, Modified British Medical Research Council questionnaire; NIV, Non-Invasive Ventilation; QMS, Quadriceps muscle strength; SGRQ, Saint George’s Respiratory Questionnaire; 1minSTS, 1-minute Sit-to-Stand Test.

^*^ p<0.05 when compared with all other profiles

^a^ p<0.05 vs Profile 1

^b^ p<0.05 vs Profile 2

^c^ p<0.05 vs Profile 3

^d^ p<0.05 vs Profile 4

**References**

1. Madhulatha TS, editor Comparison between k-means and k-medoids clustering algorithms. International Conference on Advances in Computing and Information Technology; 2011: Springer.

2. Tibshirani R, Walther G, Hastie TJJotRSSSB. Estimating the number of clusters in a data set via the gap statistic. 2001;63(2):411-23.

3. Biau G, Scornet E. A random forest guided tour. Test. 2016;25(2):197-227.
